# Identification of water content in nanocavities

**DOI:** 10.1186/1556-276X-8-171

**Published:** 2013-04-15

**Authors:** Maysoun Douas, Manuel I Marqués, Pedro A Serena

**Affiliations:** 1 Departamento de Física de Materiales C-04, Universidad Autónoma de Madrid, Madrid 28049, Spain; 2 Instituto de Ciencia de Materiales de Madrid, Consejo Superior de Investigaciones Científicas, Madrid 28049, Spain; 3 Instituto de Ciencia de Materiales ‘Nicolás Cabrera’, Universidad Autónoma de Madrid, Madrid 28049, Spain

**Keywords:** SNOM, Viral capsid, Water, FDTD, Lattice gas

## Abstract

A tapered dielectric waveguide that scans, at constant height, a sample containing a viral capsid is studied by combining a lattice gas model to simulate water meniscus formation and a finite difference time domain algorithm for light propagation through the media involved. Our results show different contrasts related to different water contents and different meniscus orientations. We propose this method as a way to study water content and evaporation process in nanocavities being either biological, like viral capsides, or nonbiological, like photonic crystals.

## Background

Hydrophilic tips used in scanning near field optical microscope (SNOM) condense some water layers, leading to the formation of a water bridge (or water meniscus) between the tip and a hydrophilic sample for small tip-sample distances. The shape of such meniscus will depend on the geometry of both surfaces, their separation, and environmental conditions (temperature and relative humidity). When working in air conditions using local probes, humidity causes characteristic jump-to-contact events due to the spontaneous formation of a water meniscus between tip and sample [1]. Presence of water at experimental relatively high humidity conditions also modifies the dielectric properties of the medium between the SNOM tip and substrate. As a consequence, the optical images of samples on surfaces are altered by humidity and water condensation. Previous studies on the optical signal under variable environmental humidity [2,3] have shown the conditional increase in the optical signal depending on the hydrophobic character of the sample. In fact, the inclusion of water condensation should be considered for any modeling or simulation of the field enhancement effect [3].

On the other hand, water condensation at the nanoscale is known to play an important role in the collapse of viral capsids during desiccation, as revealed by atomic force microscopy (AFM) experiments [4]. The meniscus formation along with the geometry of the nanocavity allows capillary force to modify the mechanical stability towards collapse [5]. An important issue that arises from these AFM studies on biological samples is whether the condensation of water in these viral nanocavities may be detected by a direct measurement.

The previously mentioned changes on the near field optics, during the desiccation stages, may be a good tool for showing how this process takes place. Indeed, SNOM characterizes sample composition by the changes in the optical near field and, since the viral capsides are almost transparent at optical wavelengths [6], different water contents in these nanocavities will produce different output signals which are distinct enough to characterize and monitor the desiccation sequence by SNOM experiments.

The aim of this paper is to understand, using an adequate combination of numerical techniques, how water evaporation or condensation in a nanocontainer (viral capsid) might be detected by near-field optic measurements. To do so, we consider a tapered dielectric waveguide that scans, at constant height, a sample formed by a viral capsid with different water contents. The manuscript is organized as follows: next section describes the system under study and the set of numerical methods we have used; finally, the two sections devoted to results and conclusions will describe the changes of the optical signal due to the presence of a water meniscus and the possible use of these changes to monitor real-time evolution of water meniscus in nanocontainers.

## Methods

### Tip-sample system

In order to describe the tip-sample system we have considered a tapered optical fiber probe, with a final aperture of 100 nm, coated with a perfect metal. This tip is placed at a constant distance, *h*=50 nm, from a flat dielectric substrate with a refractive index *n*=2.0 and 10 nm thicknesses. This geometry is very similar to that previously described by Wang *et al*[7]. Upon the substrate we have placed a simple geometry nanocontainer that simulates a viral capsid with a single porous, similar to the previously studied *ϕ*29 viral particles [4]. The considered shape of our nanocontainer is a 30-nm lateral size square with a porous of 5 nm centered at one side. The nanocontainer is almost transparent (*n*=1.06) and hydrophilic. The capsid might be filled up with double-stranded DNA (dsDNA) (refractive index *n*=1.55 at the considered wavelength) [8] or with different contents of water (*n*=1.33) that will depend on the relative humidity.

### Simulation methods

The water meniscus formation inside the container is studied using a 2D lattice gas model that has been extensively used to study water properties, including gas-liquid transition and density anomalies. This model has been also used to describe the geometry features of the water meniscus formed between an AFM tip and a substrate [9]. The fluid is represented by a 2D square lattice with a spacing of 0.3 nm. In the model, we may assume thermal and phase equilibrium with a bulk reservoir, specified by a temperature *T* and a chemical potential *μ*. These quantities are directly related to the relative humidity *R*_*h*_ through the expression *R*_*h*_=exp(*μ*−*μ*_*c*_)/*k*_B_*T*, being *k*_B_ the Boltzmann constant and *μ*_c_ the critical chemical potential. We have performed a (*V*,*T*,*μ*) Monte Carlo (MC) numerical simulation at laboratory conditions, *T*=293 K, assuming that each lattice site (*i*,*j*) was either occupied with a water molecule *ρ*(*i*,*j*)=1 (liquid phase) or empty *ρ*(*i*,*j*)=0 (gas phase). The quantity *ρ*(*i*,*j*) is the occupation number of a given site (*i*,*j*). Each water-occupied site interacts with its (occupied) neighbor sites with an attractive energy *∈* = 9 kJ/mol. This value has been chosen in order to use a model able to fit the value of the water critical temperature. The interaction of tip and nanocontainer with a water molecule involves an interaction energy given by *b*_*T*_=−56 kJ/mol (hydrophilic character). The substrate has a repulsive interaction with water given by |*bs*| = 46 kJ/mol (hydrophobic character). The conditions considered correspond to equilibrium bulk evaporation. The concrete expression of the Hamiltonian we have considered is reported in [5] and includes water-water, water-tip, and water-substrate terms. For a given set of geometrical parameters and physical conditions (temperature and humidity), an approximate shape of the water meniscus is obtained from an averaging procedure involving hundreds of different configurations. Water density average at each lattice site (0<<*ρ*(*i*,*j*)><1) was calculated after the statistical methodology described in [4]. Once <*ρ*(*i*,*j*)> was known for every site of the 2D square lattice, the effective refractive index *n*(*i*,*j*) at a given site is calculated, assuming that there is a linear dependence (*n*(*i*,*j*)=1+0.33<*ρ*(*i*,*j*)>) between the refractive index and the average water density [10]. This methodology allows to determine the meniscus shape as well as the associated refractive index map for a given set of parameters (tip-sample distance, temperature, and humidity).

The local refractive index *n*(*i*,*j*) determines the propagation of the optical signal through the tip-sample-substrate system. The propagation of the electromagnetic radiation was studied by means of a 2D finite difference time domain (FDTD) simulation, based on Yee algorithm [11]*], with a perfect matching layer as boundary condition [*[12]. Transverse Magnetic to the z direction fundamental mode is propagated through the dielectric coated fiber guide with frequency *ν*=3.77×10^15^ Hz (*λ*=500 nm). Radiated intensity, at transmission, is integrated at a plane surface, acting as light collector, located at a distance *D*=100 nm from the substrate. In our study, all intensities are normalized to that one obtained without any substrate. Since the lattice parameter used in the MC simulations is too small for being considered in feasible FDTD simulations, a larger integration lattice constant is required. In order to match FDTD lattice constant with the one used in the lattice gas simulation, a lattice step of 0.9 nm was considered for the FDTD simulations. In this way, the refractive index for each FDTD node was obtained by averaging those local refractive index values corresponding to the water nodes included within the FDTD cell. General assumptions were taken into account for the simulation. Indeed, all water necks calculated at equilibrium were considered to be stable during the typical times associated to the wave propagation; furthermore, we have neglected SNOM probe oscillations near the sample. In addition, water heating processes are not considered since radiation wavelength is far from those corresponding to water absorption bands.

## Results and discussion

**Figure 1:**
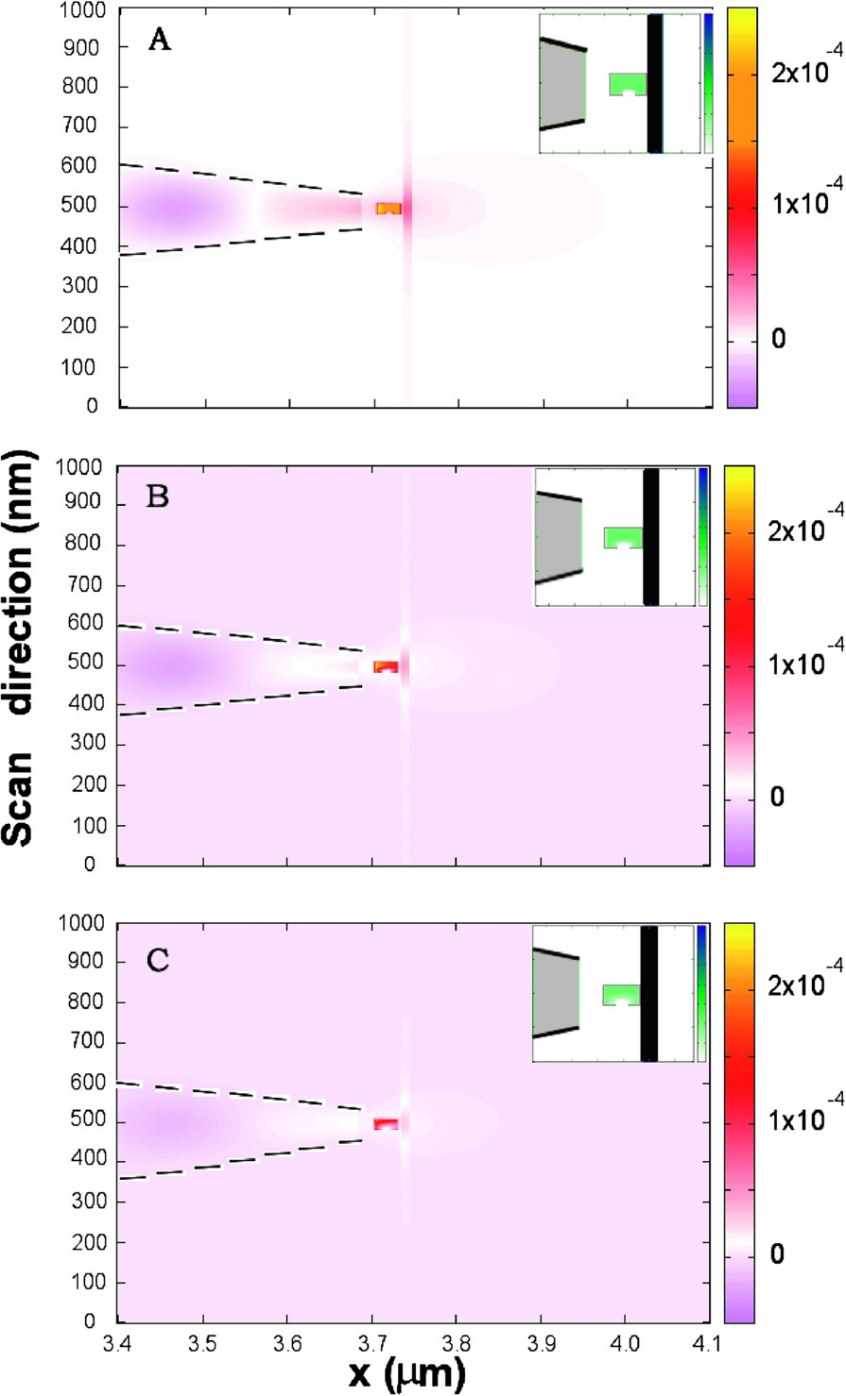
**Contribution of the water meniscus inside the viral capsid to the optical signal.** Intensity color maps at different desiccation stages are shown for values of water occupation: 100*%* (**A**), 75*%* (**B**) and 50*%* (**C**). Insets show refractive index color map showing the corresponding water density. As a guided for the eye black lines have been used to highlight tip and capsid contours.

In our first simulation we have placed the SNOM tip above the capsid and we have calculated the intensity map on our grid as a function of the water content in the nanocavity (see Figure 1). In order to highlight the effect due to the existence of water inside the nanocontainer, the background signal corresponding to the absence of any viral capsid has been subtracted. Values are normalized to the intensity source. Note how the existence of a viral capsid affects not just to the intensity in the cavity, but also to the surrounding areas and the optical fiber as well. This influence clearly depends on the nanocavity water content.

**Figure 2:**
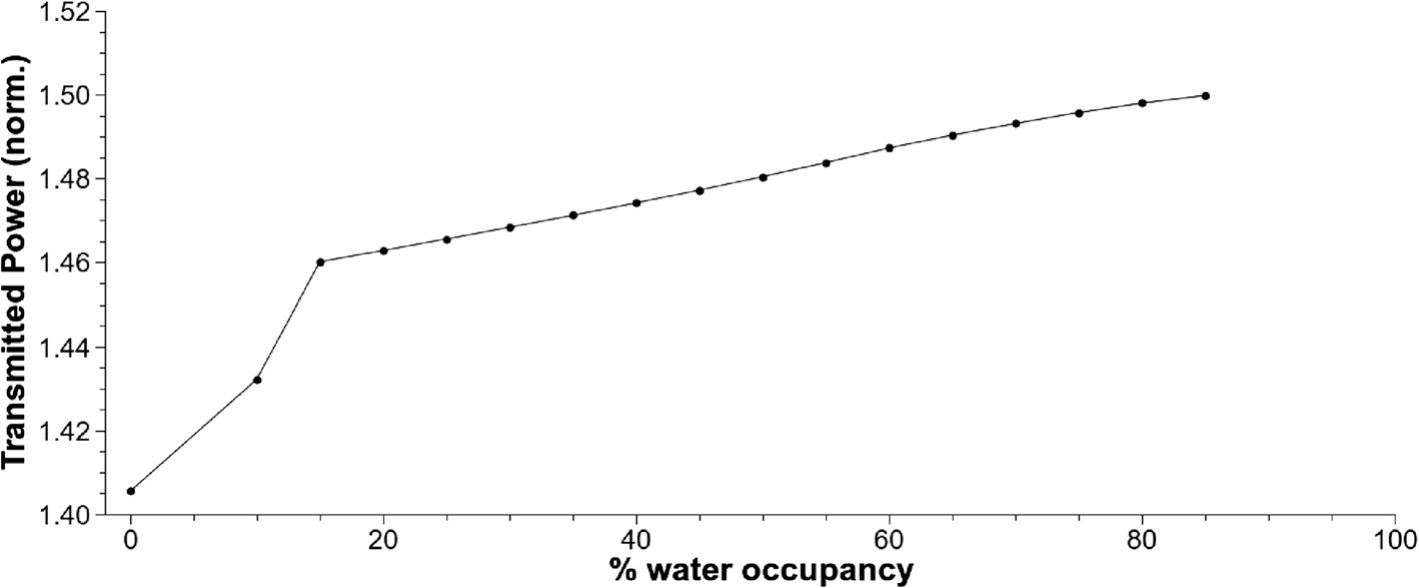
**Normalized transmitted power versus water occupancy.** Note the slope change near 15*%* of water occupancy due to the phase change inside the capsid.

In order to study the effect on the SNOM signal, we plot the total transmitted normalized intensity as a function of the water content in Figure 2. Note how desiccation affects to light intensity by decreasing the SNOM signal in a 7.5*%*. Furthermore, the change on water phase in the last stages of the desiccation process is detected by an abrupt decay of the transmitted power for values of the water occupancy close to the 15%.

**Figure 3:**
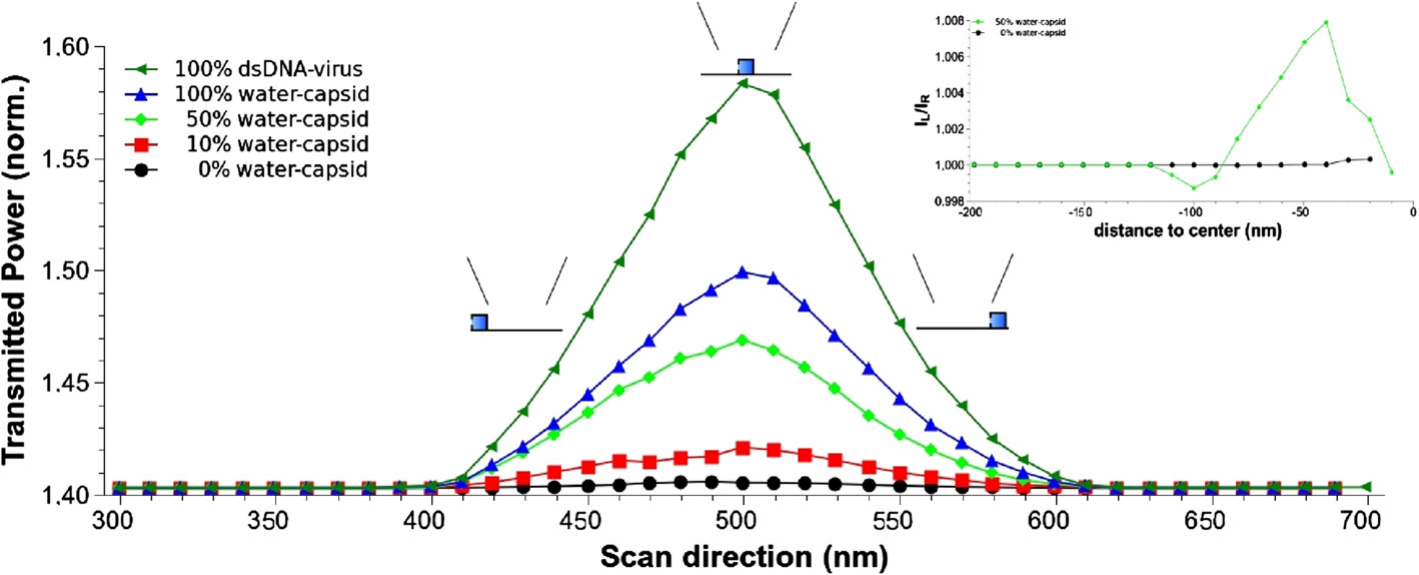
**Normalized transmitted power versus SNOM tip position over the capsid.** The calculation has been performed for the dsDNA virus (green triangles) and for empty nanocontainers with different water occupancy: 100% (blue triangles), 50% (green diamonds), 10% (red squares) and 0% (black circles). The relative position of the tip with respect to the virus capsid (represented with blue squares), for three different values of the scan direction, is shown. Inset shows the asymmetry degree in the optical signal (see text) for the empty capsid and for a container with a 50% water content.

In our second simulation, we have scanned the tip over the viral capsid and we have calculated the transmitted power for different tip positions. We have performed these simulations for different water contents and for the virus filled up with dsDNA. Results are shown in Figure 3. It is clear that SNOM scans provide capsid images that are far from its actual geometry and lateral dimensions. However, there is a clear increase in the signal for the case of the virus filled with dsDNA in the order of 15*%*. The intensity change decreases when the DNA is removed and the viral capsid is filled up with water. This change clearly depends on the water content inside the nanocontainer. Therefore, the presence of DNA or water inside the cavity clearly enhances the contrast of the container image, although it does not provide good images of the actual geometry of the sample.

There is another interesting point that must be addressed. In this specific case, we can take advantage of the signal’s broadening to study the evaporation dynamics related to meniscus geometry induced by the asymmetry porous position. This is clearly reflected by the following important feature: the power transmitted as a function of the tip position is not symmetric. This property is due to the intrinsic virus geometry, with a single porous on one side of the viral capsid implying a nonsymmetric water disposition inside the container. Interestingly, information about virus geometry as well as water evaporation dynamics may be obtained by the position of the maximum of the transmitted signal. For example, note how a porous located at the left implies a maximum on the signal displaced to the right. This asymmetry in the power is quantified in the inset in Figure 3, where the ratio between left and right transmitted signals, at equidistant points from the geometric center in the scan direction, are plotted versus distance to center. We consider an empty capsid and a container with 50% water content. Note that for the last case, a slight asymmetry shows up with a maximum value of almost 1%.

## Conclusions

We have presented a theoretical study in which we combine the lattice gas model to simulate water meniscus formation and a FDTD algorithm for light propagation through the media involved. We simulate a tapered dielectric waveguide that scans, at constant height, a sample containing a viral capsid. Our results show different contrasts related to different water contents and different meniscus orientations. We propose this method as a way to study water content and evaporation process in nanocavities being either biological, like viral capsides, or nonbiological, like photonic crystals.

## Competing interests

The authors declare that they have no competing interests.

## Authors’ contributions

MD performed the code, the calculations, and the data analysis. MIM and PAS performed data analysis. All authors contribute in formulating the manuscript. All authors read and approved the final manuscript.
